# Antimycobacterial and Nitric Oxide Production Inhibitory Activities of *Ocotea notata* from Brazilian Restinga

**DOI:** 10.1155/2015/947248

**Published:** 2015-02-19

**Authors:** Isabela Francisca Borges Costa, Sanderson Dias Calixto, Marlon Heggdorne de Araujo, Tatiana Ungaretti Paleo Konno, Luzineide Wanderley Tinoco, Denise Oliveira Guimarães, Elena B. Lasunskaia, Ivana Ramos Correa Leal, Michelle Frazão Muzitano

**Affiliations:** ^1^Laboratório de Produtos Naturais (LaProN), Curso de Farmácia, Universidade Federal do Rio de Janeiro, Campus Macaé, Polo Novo Cavaleiro-IMMT, R. Aluísio da Silva Gomes 50, 27930-560 Macaé, RJ, Brazil; ^2^Laboratório de Biologia do Reconhecer, Centro de Biociências e Biotecnologia, Universidade Estadual do Norte Fluminense Darcy Ribeiro, 28013-602 Campos dos Goytacazes, RJ, Brazil; ^3^Núcleo de Estudos em Ecologia e Desenvolvimento Sócio-Ambiental de Macaé, Universidade Federal do Rio de Janeiro, 27910-970 Macaé, RJ, Brazil; ^4^Instituto de Pesquisas de Produtos Naturais, Universidade Federal do Rio de Janeiro, 21941-902 Rio de Janeiro, RJ, Brazil; ^5^Laboratório de Produtos Naturais e Ensaios Biológicos (LaProNEB), Departamento De Produtos Naturas e Alimentos, Faculdade de Farmácia, Universidade Federal do Rio de Janeiro, 21941-902 Rio de Janeiro, RJ, Brazil

## Abstract

The genus *Ocotea* (Lauraceae) is distributed mainly in tropical and subtropical regions. Some species of this genus as *O. puberula* and *O. quixos* have been described in the literature, showing antibacterial activity. And *Ocotea macrophylla* showed anti-inflammatory activity with inhibition of COX-1, COX-2, and LOX-5. The purpose of this study was the phytochemical investigation of the plant species *Ocotea notata* from Restinga Jurubatiba National Park, Macaé, RJ, Brazil, and the search for antimycobacterial fractions and compounds. The crude extract was evaluated for antimycobacterial activity and presented 95.75 ± 2.53% of growth inhibition at 100 *µ*g/mL. Then, it was subjected to a liquid-liquid partition and subsequently was chemically investigated by HPLC, revealing the major presence of flavonoids. In this process the partition fractions hexane, ethyl acetate, and butanol are shown to be promising in the antimycobacterial assay. In addition, ethyl acetate fraction was chromatographed and afforded two flavonoids identified by MS and NMR as afzelin and isoquercitrin. The isolated flavonoids afzelin and isoquercitrin were evaluated for their antimycobacterial activity and for their ability to inhibit NO production by macrophages stimulated by LPS; both flavonoids isoquercitrin (Acet22) and afzelin (Acet32) were able to inhibit the production of NO by macrophages. The calculated IC_50_ of Acet22 and Acet32 was 1.03 and 0.85 *µ*g/mL, respectively.

## 1. Introduction

Brazilian Atlantic forest areas were considered the fourth in relevance among a total of 25 hotspots worldwide. Hotspots are areas that hold an exceptional concentration of endemic plants and vertebrates experiencing exceptional loss of habitat [1].

The plant communities at the periphery of the Atlantic rainforest complex, such as restingas, differ from the core formation in that they exhibit more extreme environmental conditions found in these systems; drought, salinity, high temperatures, and low soil nutrient contents are the main limiting factors in the open scrub habitat of the restinga vegetation [2].

*Ocotea notata* (Nees & Mart.) Mez, Lauraceae, is a medium sized tree, popularly known as white cinnamon. The genus is distributed throughout tropical and subtropical regions, especially along the Brazilian coast.* Ocotea* species have been studied for their diversity in secondary metabolites such as alkaloids, neolignans, lignans, terpenes, and flavonoids [3–8] and stand out for their biological activities such as anti-inflammatory [9, 10], antioxidant [11, 12], antiprotozoan [13], antiallergic [14], central nervous system depressant [15], antimicrobial [11, 16], and anti-herpetic [8].

The present study aims at the investigation of the chemical profile of* Ocotea notata* ethanol extract collected in Jurubatiba Restinga (Macaé, RJ, Brazil) through HPLC analyses and phytochemical study. In addition, due to the previously described antimicrobial activity of* O. notata*, the antimycobacterial activity of the samples obtained in this chemical study and their ability to inhibit NO production by macrophages stimulated by LPS were also investigated.

## 2. Materials and Methods 

### 2.1. General Experimental Procedures

^1^H and ^13^C-NMR spectra were recorded on a Bruker DRX-400 NMR spectrometer (^1^H: 400 MHz; ^13^C: 100 MHz). Chromatography was performed on reversed-phase silica Kieselgel 60 silanisiert (0,063–0,200 mm). Eluates were monitored by thin-layer chromatography (TLC) on silica 60 F_254_ (Merck) using butanol/acetic acid/water (BAW 8 : 1 : 1), visualized under UV light and revealed with NP-PEG.

### 2.2. Botanical Material

The plant species studied was collected in Restinga de Jurubatiba National Park, Quissamã (southeastern Rio de Janeiro state, Brazil): 22.19828°S; 41.46338°W. Voucher specimens were deposited at the Herbarium of the Rio de Janeiro Federal University, Brazil (RFA38751), after identification by the botanist Tatiana U. P. Kono. This research was complied with all relevant federal guidelines and institutional policies related to the botanical material for research purposes.

### 2.3. Ethanol Extraction and Partitions

Fresh leaves (1,300.00 g) were triturated and extracted exhaustedly with ethanol ACS at room temperature (crude extract). An aliquot (60 g) of the dried extract (78.07 g) was resuspended with methanol and partitioned with hexane to obtain the hexane fraction (26.68 g). Methanol residual phase was dried and resuspended with pure water and partitioned sequentially with ethyl acetate and butanol, affording ethyl acetate fraction (3.40 g) and butanol fraction (10.50 g), respectively. The residual aqueous phase was named the aqueous fraction (13.99 g). An aliquot of ethyl acetate fraction (1.50 g) was chromatographed on a silica Kieselgel 60 silanisiert (0,063–0,200 mm) (H_2_O/MeOH gradient), yielding 295 fractions. Similar fractions between 92–96 (Acet22) and 166–168 (Acet32) were grouped observing the chromatographic similarities, after TLC revealed with NP-PEG solution with 1% ethanolic diphenylboryloxy-ethylamine acid (NP) followed by 5% polyethylene glycol-4000 (PEG) with 10 mL and 8 mL, respectively. By TLC analysis, it was possible to observe a single spot in these subfractions, suggesting the purity of the sample.

### 2.4. Analysis by High-Performance Liquid Chromatography (HPLC)

Extracts, fractions, and isolated compounds were submitted to high-performance liquid chromatography using a Shimadzu Prominence LC-20. The detection was performed at fixed wavelengths of 254 nm and 332 nm. The column used was a Nucleosil 100-5 RP-18 (5 mM, 4.0 × 250 mm) maintained at 30°C. The eluents were (A): H_2_O adjusted to pH 3.0 by H_3_PO_4_ and (B): CH_3_CN. The following solvent gradients (v/v) were applied: from H_2_O (pH 3.0)-CH_3_CN (10 : 0) to H_2_O (pH 3.0)-CH_3_CN (8.5 : 1.5) within 10 min; from H_2_O (pH 3.0)-CH_3_CN (7.5 : 2.5) to H_2_O (pH 3.0) within 30 min-CH_3_CN (5 : 5) within 45 min; from H_2_O (pH 3.0)-CH_3_CN (0 : 10) to H_2_O (pH 3.0) within 60 min; from 60 minutes as total time of analysis. Flow elution was 1 mL/min^−1^ and 10 *μ*L samples were injected. HPLC analyses were performed after dilution of 10 mg of the extract in 1 mL of purified water.

### 2.5. Quantification of Flavonoids

The flavonoid quantification was carried out using calibration graph with ten data points. Calibration graph for HPLC was recorded with rutin (quercetin 3-O-rutinoside) amounts ranging from 0.20 to 10.0 *μ*g. The linearity range of the detector response was verified using a series of twofold diluted solutions of rutin. The relationship between peak areas (detector responses) and amount of rutin was linear over 1000–20 *μ*g/mL (*r* ^2^ = 0.9999). To evaluate the repeatability of the injection integration, the rutin standard solution and the extract were injected three times and the relative standard deviation values were calculated.

### 2.6. Antimycobacterial Activity of the Extract, Fractions and Isolated Compounds

All samples were evaluated for their antimycobacterial activity, at concentrations of 0.8, 4, 20, and 100 *μ*g/mL, using a tetrazole salt assay in 96-well microplate [17]. Initially, 300 *μ*L of a suspension of* Mycobacterium bovis* BCG strain Moreau (3 × 10^7^ CFU/mL) was incubated with 7.2 mL of Middlebrook 7H9 medium supplemented with 0.05% Tween 80 and albumin-dextrose-catalase (ADC). When at logarithmic growth phase, 50 *μ*L was plated in a 96-well microplate at 1 × 10^6^ CFU/well, and 50 *μ*L of each sample was added in four concentrations. The sealed microplate was incubated at 37°C and 5% CO_2_ for 7 days. After this period, 10 *μ*L of tetrazolium salt (3-[4,5-dimethylthiazol-2-yl]-2,5-diphenyltetrazole, 5 mg/mL in sterile PBS) was added, and after 3 hours 100 *μ*L of lyses buffer (20% w/v sodium dodecyl sulfate, SDS/50% dimethylformamide, DMF in distilled water, pH 4.7) was added. The microplate was incubated overnight, and the reading was made using a spectrophotometer at 570 nm. As a positive control, a culture medium with bacteria and the antibiotic rifampin (Sigma-Aldrich, 95% purity), at concentrations of 0.0011, 0.0033, 0.01, and 0.03 *μ*g/mL, were used. As a negative control, a culture medium with bacteria untreated with the samples was used. The test was performed in triplicate and the average value and standard deviation were calculated.

### 2.7. Determination of Nitric Oxide Production by Macrophage RAW 264.7 and Cytotoxicity

The murine macrophage cell line RAW 264.7 was obtained from the American Type Culture Collection (ATCC) and grown at 37°C and 5% CO_2_ in DMEM F-12 that was supplemented with 10% FCS and gentamicin (50 *μ*g/mL). RAW 264.7 cells (1 × 10^5^ cells/well) were seeded in flat bottom 96-well tissue culture plates (Corning, Inc.) in the presence or absence of four concentrations of the samples (0.8, 4, 20, and 100 *μ*g/mL) and/or LPS (*Escherichia coli* 055:B5; Sigma-Aldrich). After a 24-hour incubation period, culture supernatants were collected and nitrite, a stable NO metabolite, was determined by using the Griess test [18]. As a positive control, macrophages untreated but stimulated with 1 *μ*g/mL LPS were used. As a negative control, macrophages not treated and not LPS-stimulated were used. A nitric oxide synthase inhibitor, NG-methyl-L-arginine acetate salt (L-NMMA, Sigma-Aldrich, 98% purity), was also used as a positive control at 20 *μ*g/mL inhibiting 59.22 ± 2.96% NO production. The release of cytoplasmic enzyme lactate dehydrogenase (LDH) was determined using 50 *μ*L of culture supernatant collected at the end of the assay [19]. The LDH content, which represents an indirect indication of cytotoxicity, was determined colorimetrically using a commercial kit (Doles). The specific release was calculated as a percentage of the controls (untreated macrophages as the negative control and 1% Triton X-100 [Vetec Chem.] detergent treated macrophages as the positive control). Final concentrations of DMSO, used as the solvent of the samples, were tested in parallel as a control. Tests were performed in triplicate, and the mean value and standard deviation were calculated.

### 2.8. Statistical Analysis

All experiments were performed in triplicate and results were expressed as mean ± standard deviation (M ± SD). Data was evaluated by one-way ANOVA followed by Tukey test and considered statistically significant for *P* < 0.05. The IC_50_ (concentration able to modulate at 50% maximum activity) of the samples tested was calculated by nonlinear regression using the results of the concentration-response curves. Microsoft Office Excel and GraphPad Prism software were used.

## 3. Results and Discussion

**Figure 1:**
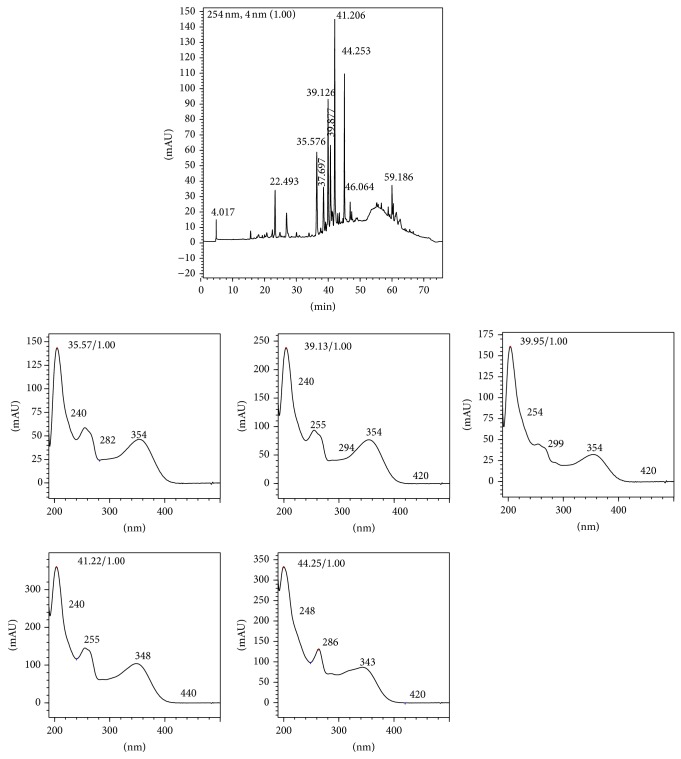
Chromatogram at 254 nm of the crude extract of the leaves of* Ocotea notata* by HPLC. Emphases for the UV spectrum of peaks 35.57, 39.13, 39.88, 41.22, and 44.25 min.

**Figure 2:**
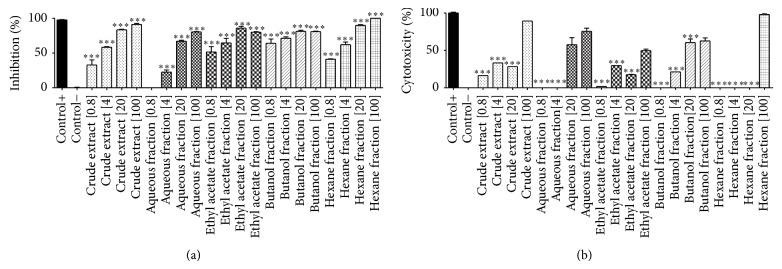
(a) Inhibitory effect of* Mycobacterium bovis* BCG growth by extracts and fractions of leaves of* Ocotea notata* tested at four different concentrations, 0.8, 4, 20, and 100 *μ*g/mL. The percentage of inhibition was compared with the positive control (rifampicin): 98.17 ± 0.8%/O.D.: 0.180 ± 0.8 and negative control (mycobacteria without treatment) 0.1 ± 0.1%/O.D.: 0.880 ± 1.24. (b) Cytotoxicity of the extracts and fractions of leaves of* Ocotea notata* tested at four different concentrations 0.8, 4, 20, and 100 *μ*g/mL in murine macrophages RAW 264.7. The percentages were compared to positive control, macrophages treated with Triton 1%: 98.88 ± 1.6/O.D.: 0.132, and negative control, macrophages without treatment 0.01 ± 0.1/O.D.: 0.749. ^∗^ *P* < 0.05, ^∗∗^ *P* < 0.01, and ^∗∗∗^ *P* < 0.001, significance obtained by ANOVA and posttest Tukey (*n* = 3), when compared to the negative control (a) and positive control (b).

*Ocotea notata* ethanol extract (crude extract) was analyzed by reversed-phase HPLC-DAD to study its chemical profile. A suitable methodology was developed and five major peaks were identified with retention time of 35.57, 39.13, 39.88, 41.22, and 44.25 minutes (Figure 1). UV spectrum of each peak revealed the flavonoid absorption profile (typical *λ*_max⁡_ 251–271 and 335–350 nm) [20]. For the predominance of flavonoids in the sample, they were quantified based on an area  ×  *μ*g calibration curve obtained using a rutin external standard. The sum of all identified peaks in the chromatogram was assumed to represent the total flavonoid content in the extract, expressed as rutin equivalents, percentage (w/w) g/100 g of crude extract. For this purpose, crude extract was analyzed in triplicate resulting in a flavonoid content of 2.71 ± 0.16% w/w. Crude extract was assessed in view of verifying its antimycobacterial activity. Antimycobacterial activity was evaluated on* Mycobacterium bovis* BCG strain. This strain shows a very similar genetic profile when compared to* M. tuberculosis* [21].* Ocotea notata* crude extract exhibits, at concentration of 20 *μ*g/mL, 73.63 ± 1.86% of mycobacterial growth inhibition and only 26.40 ± 1.50% of cytotoxicity (Figures 2(a) and 2(b)). At concentration of 100 *μ*g/mL, it showed an inhibition of 95.75 ± 2.53%, but it was toxic when evaluated in RAW 264.7 macrophages culture (Figures 2(a) and 2(b)). The inhibition extract capacity was compared to rifampicin, drug tested at different concentrations, and used as a positive control. Tuberculosis (TB) is one of the leading causes of mortality worldwide and its etiologic agent is* Mycobacterium tuberculosis* bacilli but also* M. bovis*,* M. africanum*, and* M. microti* [22].

For the promising activity observed from the crude extract, it was fractioned by liquid-liquid partition and afforded four fractions with different polarities, hexane, ethyl acetate, butanol, and water. The fraction that had the best performance in the inhibitory mycobacterial activity growth was the hexane fraction. At concentrations of 0.8, 4, 20, and 100 *μ*g/mL it showed, respectively, 41.63 ± 0.80%; 65.75 ± 5.30%; 90.88 ± 1.59; and 102.56 ± 1.90% of mycobacterial growth inhibition. Hexane fraction showed activity even in small concentrations and it was toxic for macrophages only at the highest concentration. This finding suggested selectivity for antimycobacterial activity without being cytotoxic to macrophages at 0.8, 4 and 20 *μ*g/mL. This fraction is the most apolar and usually this kind of fraction is mainly composed by terpenes, sterols, and fatty acids [23]. Hexane fraction was followed by ethyl acetate fraction that was the second on inhibition of mycobacterial growth.

**Figure 3:**
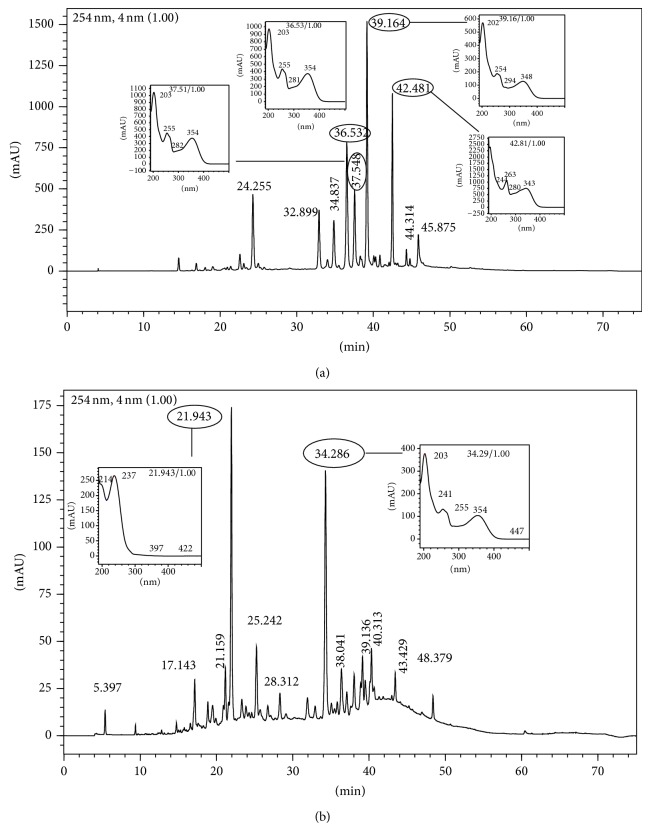
(a) Chromatogram at 254 nm of ethyl acetate fraction from leaves of* Ocotea notata* by HPLC. Emphases for the UV spectrum of peaks 36.53; 37.55; 39.16; and 42.48 min. (b) Chromatogram at 254 nm of butanol fraction from leaves of* Ocotea notata* by HPLC. Emphases for the UV spectrum of peaks 21.943 and 34.286 min.

The inhibitory activity of the ethyl acetate fraction was at concentrations of 0.8, 4, 20, and 100 *μ*g/mL, 43.63 ± 1.06, 57.75 ± 0.46, 83.38 ± 3.54, and 80.75 ± 1.15%, respectively. But when the cytotoxicity to macrophages was evaluated, the ethyl acetate fraction showed low toxicity when compared to hexane fraction at the highest concentration (Figure 2). The same was observed in butanol fraction (Figure 2). According to Moresco and Brighente [24] fractions as ethyl acetate, butanol, and aqueous are rich in phenolic compounds; this can be explained by the polarity of these substances. Comparing the demonstrated results, it was noticed that butanol and ethyl acetate fractions showed an excellent inhibitory effect and lower cytotoxicity, especially the last one, so that these polar fractions were investigated by HPLC to identify the chemical constituents responsible for this activity. HPLC profile of polar fractions pointed the presence of secondary metabolites such as flavonoids (Figures 3(a) and 3(b)). Butanol fraction presented two major peaks, 21.94 and 34.29 min at 254 nm, the second one with UV flavonoid characteristic. Ethyl acetate fraction showed a complex profile with four major peaks that were identified by UV as flavonoids (36.53, 37.55, 39.16, and 42.48 min). Ethyl acetate fraction was chosen by fractionation although butanol and hexane fractions were also selected except for future investigations. To compare the total flavonoid content of the ethyl acetate fraction and the crude extract, as reported above, this fraction was analyzed in triplicate resulting in a flavonoid content of 37.3 ± 1.5% w/w, rate over fifteen times higher than that found in crude extract.

**Figure 4:**
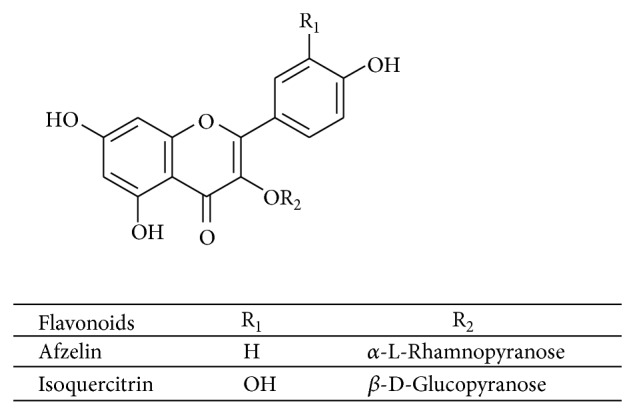
Kaempferol 3-*O*-*α*-L-rhamnopyranoside (afzelin-Acet32), quercetin 3-*O*-*β*-D-glucopyranoside (isoquercitrin-Acet22).

Reversed-phase chromatography of ethyl acetate fraction afforded two isolated flavonoids codified as Acet22 and Acet32. These two flavonoids were analyzed by HPLC and their purity was confirmed. Mono and bidimensional ^1^H and ^13^C NMR analyses of Acet22 allow this flavonoid characterization as isoquercitrin (quercetin 3-*O*-*β*-D-glucopyranoside) (Figure 4). NMR data are in accordance with the literature [25]. The flavonoid Acet32 was analyzed by NMR and MS. The molecular formula C_21_H_20_O_10_ was deduced from the molecular ion* m/z* 455.2 [M + Na–H]^+^ (calculated for C_21_H_20_O_10_Na). Analyzing the molecule fragmentation pattern can be observed in the following peaks representing the fragments [M-Ram + Na]^+^.* m/z*  (309.0) relative to the loss of rhamnose residue and [M-Kaempferol + Na]^+^. (*m/z* 169.1) relative to the loss of the aglycone. These results together with NMR data allow the identification of the flavonoid afzelin (kaempferol-3-*O*-*α*-L-rhamnopyranoside) (Figure 4) as Acet32, in accordance with literature [25].

**Figure 5:**
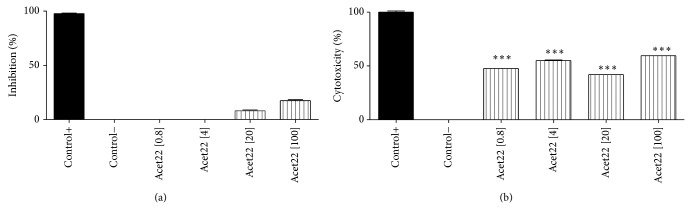
(a) Inhibitory effect of* Mycobacterium bovis* BCG growth by Acet22 (isoquercitrin) tested at four different concentrations, 0.8, 4, 20, and 100 *μ*g/mL. The percentage of inhibition was compared with the positive control (rifampicin), 98.17 ± 0.8%/O.D.: 0.180 ± 0.8, and negative control (medium + mycobacteria), 0.1 ± 0.1%/O.D.: 0.880 ± 1.24. (b) Cytotoxicity of Acet22 (isoquercitrin) tested at four different concentrations 0.8, 4, 20, and 100 *μ*g/mL in murine macrophages RAW 264. The percentages were compared to positive control, macrophages treated with Triton 1%: 98.88 ± 1.6/O.D.: 0.132, and negative control, macrophages without treatment 0.01 ± 0.1/O.D.: 0.749. ^∗^ *P* < 0.05, ^∗∗^ *P* < 0.01, and ^∗∗∗^ *P* < 0.001, significance obtained by ANOVA and posttest Tukey (*n* = 3), when compared to the negative control (a) and positive control (b).

**Figure 6:**
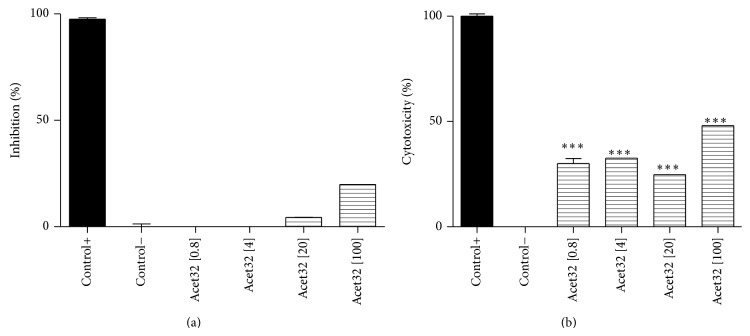
(a) Inhibitory effect of* Mycobacterium bovis* BCG growth by Acet32 (afzelin) tested at four different concentrations, 0.8; 4; 20; and 100 *μ*g/mL. The percentage of inhibition was compared with the positive control (rifampicin), 98.17 ± 0.8%/O.D.: 0.180 ± 0.8, and negative control (medium + mycobacteria), 0.1 ± 0.1%/O.D.: 0.880 ± 1.24. (b) Cytotoxicity of Acet32 (afzelin) tested at four different concentrations 0.8; 4; 20; and 100 *μ*g/mL against murine macrophages RAW 264. The percentages were compared to positive control, macrophages treated with Triton 1%: 98.88 ± 1.6/O.D.: 0.132, and negative control, macrophages without treatment: 0.01 ± 0.1/O.D.: 0.749. ^∗^ *P* < 0.05, ^∗∗^ *P* < 0.01, and ^∗∗∗^ *P* < 0.001, significance obtained by ANOVA and posttest Tukey (*n* = 3), when compared to the negative control (a) and positive control (b).

As can be seen, the isolated compounds, isoquercitrin (Figures 5(a) e 5(b)) and afzelin (Figures 6(a) e 6(b)), showed no antimycobacterial activity and moderated cytotoxicity. In the literature there are few reports about flavonoids with antimycobacterial activity. Yet some of them, especially those that are less polar, could be found, as chalcones [26] and prenylated flavones [27]. However isoquercitrin and afzelin are glycosylated flavonoid with considerable hydrophilicity. This fact complicates the permeability of these substances through lipophilic bacterial wall.

For the genus* Ocotea* there are few records about flavonoids isolation, and the main secondary metabolites are alkaloids, lignans, and terpenoids. Flavonoids are divided into classes according to their chemical and biosynthetic characteristics and have numerous pharmacological and biochemical effects [28]. There is only one report on the isolation of the flavonoid isoquercitrin from* O. notata*, in addition to a proanthocyanidin trimer and flavonoids quercitrin and reynoutrin [8]. There are reports about isoquercitrin isolation also from* O. corymbosa* [29]. No data describing the antimicrobial activity of isoquercitrin were found.

Funasaki [30] reported the phytochemical study of* O. catharinensis* leaves, describing the glycosylated flavonoid afzelin isolation, the same that is isolated and described in this study, for* O. notata*. Afzelin showed no antimicrobial activity described in the literature. But it presents antinociceptive and anti-inflammatory activities [31] and strong neural protective effect and antioxidant [32].

In addition, considering that* Ocotea notata* isolated flavonoids, isoquercitrin, and afzelin do not show significant antimycobacterial activity, as demonstrated in the present study, they were evaluated to verify their capacity of inhibiting the NO production by LPS-stimulated macrophages. Nitric oxide is a chemical mediator with microbicide activity that is produced by activated phagocytes during inflammation [33]. Inflammation is strongly involved in the pathogenesis of most infectious diseases, including tuberculosis [34]. In general, the production of proinflammatory mediators by the infected macrophages, such as IL-1*β*, TNF-*α*, and NO, is essential for protection against mycobacteria [35]. However, tissue concentrations of NO required for microbicide action are toxic to the host cells and must be tightly regulated [33]. In the case of TB most severe forms, additional anti-inflammatory therapy to prevent excessive inflammation could be required [35]. In report about treatment of TB together with anti-inflammatory drugs it was demonstrated that corticosteroids can be effective in reducing mortality for all forms of TB [36]. The benefits of anti-inflammatory treatment have also been demonstrated in some TB cases using nonsteroidal anti-inflammatory drugs (NSAIDs). Result with infected animals and treatment with ibuprofen (anti-inflammatory drug) showed decrease in pulmonary infiltrates and in bacterial load and increased survival, compared to untreated animals [37].

**Figure 7:**
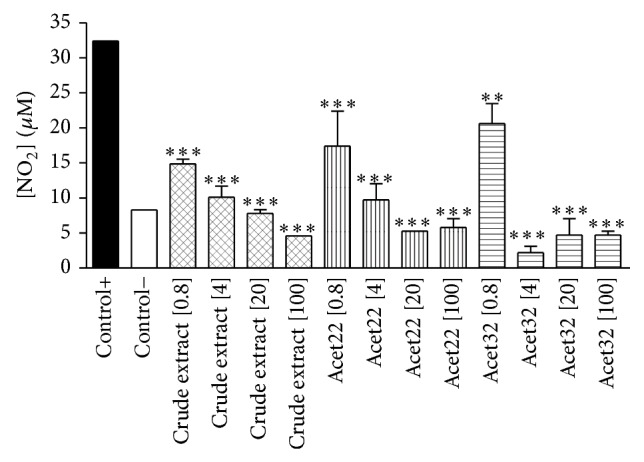
The inhibitory effect on NO production by crude extract and flavonoids Acet22 (isoquercitrin) and Acet32 (afzelin) at concentrations of 0.8, 4, 20, and 100 *μ*g/mL in lipopolysaccharide- (LPS-) stimulated RAW 264.7 macrophages. Negative control: macrophages without stimuli. Positive control: macrophages stimulated with 1 *μ*g/mL LPS. Treatment with L-NMMA was used also as a positive control of NO inhibition, reducing NO production by 59.22 ± 2.96% at 20 *μ*g/mL. ^∗^ *P* < 0.05, ^∗∗^ *P* < 0.01, and ^∗∗∗^ *P* < 0.001, significance obtained by ANOVA and posttest Tukey (*n* = 3) when compared to the positive control.

As could be seen in Figure 7, crude extract and both flavonoids, isoquercitrin (Acet22) and afzelin (Acet32), were capable of inhibiting the NO production by macrophages, with *P* < 0.001 at concentrations of 0.8, 4, 20, and 100 *μ*g/mL, when compared with positive control (LPS-stimulated RAW 264.7 macrophages). The calculated IC_50_ of crude extract, Acet22, and Acet32 was 3.24, 1.03, and 0.85 *μ*g/mL, respectively. Although the inhibitory effect in NO production was slightly associated with moderated cytotoxicity (Figures 5(b) and 6(b)), especially for isolated flavonoids, the capacity of inhibiting NO production is higher when compared to cytotoxicity. For example, at 20 *μ*g/mL, for all tested samples, NO inhibitory activity was greater than 90%, while cytotoxicity was between 20 and 40%.

## 4. Conclusion

The present study reported for the first time the antimycobacterial and NO production inhibitory activities of* O. notata* extract. The findings from this study reveal the potential of* O. notata* extract and fractions to afford bioactive compounds and suggest that afzelin and isoquercitrin isolated do not contribute to the ethyl acetate fraction activity. But these compounds were able to significantly suppress the production of NO stimulated by LPS in RAW 264.7 macrophages. Further studies have been done to better understand the reported activities, as well as the compounds' contribution to them.
